# MEWpy: a computational strain optimization workbench in Python

**DOI:** 10.1093/bioinformatics/btab013

**Published:** 2021-01-18

**Authors:** Vítor Pereira, Fernando Cruz, Miguel Rocha

**Affiliations:** Centre of Biological Engineering, University of Minho, Braga 4710-057, Portugal; Centre of Biological Engineering, University of Minho, Braga 4710-057, Portugal; Centre of Biological Engineering, University of Minho, Braga 4710-057, Portugal

## Abstract

**Summary:**

Metabolic Engineering aims to favour the overproduction of native, as well as non-native, metabolites by modifying or extending the cellular processes of a specific organism. In this context, Computational Strain Optimization (CSO) plays a relevant role by putting forward mathematical approaches able to identify potential metabolic modifications to achieve the defined production goals. We present MEWpy, a Python workbench for metabolic engineering, which covers a wide range of metabolic and regulatory modelling approaches, as well as phenotype simulation and CSO algorithms.

**Availability and implementation:**

MEWpy can be installed from PyPi (*pip install mewpy*), the source code being available at https://github.com/BioSystemsUM/mewpy under the GPL license.

## 1 Introduction

Constraint-Based Modelling (CBM) provides tools for the integrative analysis of molecular systems and quantitative prediction of physicochemical and biochemical phenotypic states. Recently, several modelling approaches have arisen putting forward a growing integration of the transcriptional and translational layers and respective omics data (e.g. transcriptomics, proteomics) with Genome-Scale Metabolic Models (GSMMs), to improve the characterization of cell physiology, while contributing to a better understanding of the organisms’ metabolism. Some illustrative approaches are the GECKO toolbox (Sanchez et al., 2017) and OptRAM (Shen et al., 2019), which respectively integrate proteomics and transcriptional regulation for enhanced phenotype predictions. These integrative modelling approaches provide computational interfaces to run phenotype prediction methods, which may be explored by Computational Strain Optimization (CSO) methods.

CSO consists on identifying the set of genetic modifications, to be introduced in an organism, that optimize a desired engineering goal. Typically, the goal is to maximize the production of a compound of interest, while assuring that the organism remains viable. Deterministic approaches to CSO problems, such as OptKnock (Burgard et al., 2003), identify the best set of genetic modifications by converting a bilevel mixed integer linear formulation into a single level one (Maia et al., 2016). While an inner problem addresses the biological objective, cellular growth, the outer problem focuses on the engineering goal, the overproduction of the desired compound. Such approaches, however, do not scale well with larger models or higher number of perturbations. Hence, alternative approaches consider meta-heuristics, such as Evolutionary Algorithms, to explore the high dimensionality search space of genetic perturbations (Rocha et al., 2008). OptFlux (Rocha et al., 2010) (written in Java) and CAMEO (Cardoso et al., 2018) (a Python library) are two open-source software frameworks, which include heuristic-based CSO, but are currently restricted to the use of GSMMs only containing metabolites, reactions and gene-protein-reaction (GPR) associations.

In this context, and given the lack of integrative tools for the increasing number of modelling approaches, we propose MEWpy, an integrated Metabolic Engineering Workbench written in Python, that offers methods to explore different classes of constraint-based models, including metabolic, enzymatic or regulatory constraints. MEWpy enables using different modelling approaches, such as the GECKO toolbox and OptRAM algorithm, to run different phenotype prediction algorithms, and allowing them to be used to support strain optimization.

## 2 Architecture of MEWpy

**Fig. 1.**
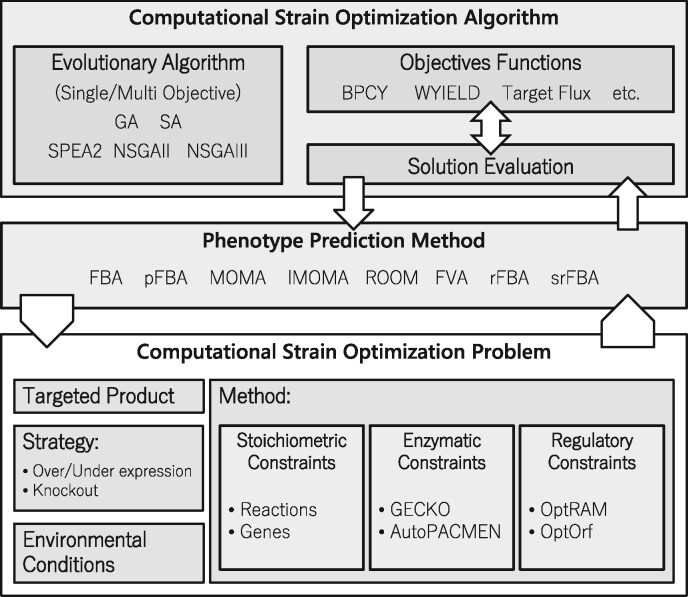
Conceptual architecture of the MEWpy framework. Phenotype prediction methods: Flux Balance Analysis (FBA); parsimonious FBA (pFBA); Minimization of Metabolic Adjustment (MOMA); linear version of MOMA (lMOMA); Regulatory On/Off Minimization of metabolic flux (ROOM); Flux Variability Analysis (FVA); regulatory FBA (rFBA); Steady-state regulatory FBA (srFBA). Evolutionary Algorithms: Genetic Algorithm (GA); Simulated Annealing (SA); Strength Pareto EA (SPEA2); Non-dominated Sorting GA, versions II (NSGAII) and III (NSGAIII). Objective functions: Biomass-Product Coupled Yield (BPCY); Weighted Yield (WYIELD)

MEWpy aims to provide a Python implementation of CSO algorithms, which can run over GSMMs defining GPR associations, but also over the previously discussed enhanced modelling approaches. The conceptual architecture of MEWpy, which is highlighted in Figure 1, encompasses three layers, from bottom to top, a problem definition layer, a phenotype simulation layer and an optimization layer, next further detailed:

- Problem definition layer. The definition of the CSO problem, includes the selected modelling framework, the definition of the modification targets (reactions, genes, proteins or regulatory variables present in the model), the modification strategy (deletion, over-/under expression), together with the target product and the environmental conditions;
- Phenotype simulation layer. This layer aggregates the methods used to evaluate the mutant strains generated by the CSO algorithm, specifically, Flux Balance Analysis (FBA), parsimonious FBA (pFBA), Regulatory On/Off Minimization of metabolic flux (ROOM), Minimization of Metabolic Adjustment (MOMA) and its linear version (lMOMA) as well as Flux Variability Analysis (FVA). MEWpy also includes implementations of the regulatory FBA (rFBA) and of the Steady-state rFBA (srFBA) to incorporate transcriptional regulation.
- Optimization layer. This layer encompasses the optimization heuristics used for strain optimization and respective objective functions. At each iteration of the optimization algorithms, the fitness of candidate solutions is asserted by running the phenotype simulations required by the objective functions.

### 2.1 Optimization algorithms

Regarding the CSO algorithms, MEWpy resorts to Evolutionary Algorithms (EAs), given their flexibility in the definition of objective functions. EAs are stochastic algorithms inspired by nature. They maintain a population of solutions, encoded metabolic modifications, whose interactions drive the optimization process. At each generation, mating and mutation operators produce a new solution set, from which the fittest are selected to integrate the next population. Such a meta-heuristic mimics the Darwinian evolutionary principles to find sets of modifications whose phenotype best address the optimization problem.

EAs have been applied in metabolic engineering frequently considering a single optimization objective or a weighted aggregated sum of distinct objective functions. Such approaches add increased difficulties: the trade-offs between optimization objectives need to be adequately chosen beforehand; each objective value needs to be normalized; single objective EAs, and in particular Genetic Algorithms (GAs), are more prone to get stuck in a local optimum, evidencing premature convergence, as they manifest more difficulty in preserving high diversity within the populations (Pandey et al., 2014).

Multi-objective EAs (MOEAs), on the other hand, enable the formulation of strain design problems that account for the simultaneous optimization of more than one objective (e.g.: product rate, growth rate, biomass product coupled yield, number of modifications). MOEAs deliver in a single run a set of solutions with different trade-offs between the objectives, providing a broader set of possible perturbations for analysis.

Currently, the EAs are implemented by the Inspyred (Tonda, 2020) and JMetalPy (Benítez-Hidalgo *et al.*, 2019) Python libraries.

### 2.2 Modelling approaches

MEWpy offers means for the exploitation of constraint-based models that account for the following types of constraints:

- Metabolic Constraints: MEWpy enables to evaluate phenotypes resulting from genes’ or reactions’ over- or under-expression, as well as deletion. Modifications of gene expression are reflected into the catalysed reactions by converting gene-protein-reaction (GPR) rules into flux constraints. GPR rules are converted to algebraic expressions replacing the (AND, OR) Boolean operators by (*min*, *max*) functions (this may be overridden according to user preferences) and gene identifiers by expression values. Modifications on reactions fluxes are achieved by altering their bounds.
- *Enzymatic constraints*: MEWpy provides tools to modify enzymatic expression. GSMMs model metabolism and gene-reaction interactions, but are oblivious to other important factors (e.g. enzyme kinetics and abundance) which affect cells’ metabolism. The incorporation of such elements as additional constraints leads to better and more accurate phenotype prediction. As such, MEWpy offers means to realize strain optimization by imposing enzymatic constraints using GECKO models (GSMM with enzymatic constraints using kinetic and omics data) (Sanchez *et al.*, 2017) or sMOMENT (short MetabOlic Modelling with ENzyme kineTics) (Bekiaris and Klamt, 2020) models.
- *Regulatory constraints*: the complex cross talking mechanisms between gene regulation and metabolism are not captured by GSMMs alone. Ergo, MEWpy also contemplates CSO strategies towards designs that impose regulatory constraints, notably, the OptORF (Kim and Reed, 2010) and OptRAM (Shen *et al.*, 2019) algorithms. The MEWpy implementation of OptORF presently allows for the identification of gene deletions (for both metabolic genes and transcription factors). On the other hand, OptRAM considers up- and down-regulation strategies, as well as deletions. While OptRAM authors propose a Simulated Annealing (SA) algorithm to identify strategies that increase the production of specific compounds in yeast (single objective), MEWpy also supports multi-objective optimization with the already mentioned added benefits.

Different phenotype simulation methods are seamlessly provided by COBRApy (Ebrahim et al., 2013) and REFRAMED libraries, including Flux Balance Analysis, and several variants adapted for the prediction on mutant phenotypes and encompassing regulatory constraints (Fig. 1).

## 3 Working examples and documentation

MEWpy globally defines optimization tasks as problems that differ on modification targets and strategy, but they all follow the same required minimal steps: (i) load a model, (ii) choose the optimization objectives, (iii) instantiate the problem and (iv) run the optimization. Next, a minimal example is presented, without the necessary imports, that aims to optimize the yield of a target product by modifying gene expression. The objective functions, to be maximized, are the biomass-product coupled yield (BPCY) and the weighed sum of the minimum and maximum product fluxes (WYIELD). 

f1 = BPCY(biomass_id, product_id, method=’lMOMA’)

f2 = WYIELD(biomass_id, product_id)

problem = GOUProblem(model , [f1, f2])

ea = EA(problem)

ea.run()

In addition, configurations may be added in steps (ii)–(iv) reflecting, for example, the chosen growth medium, a maximum number of allowed modifications, the selected EA, the number of iterations or the number of parallel threads. The MEWpy documentation, which can be found at https://mewpy.readthedocs.io, covers the extensive list of available configurations. Additionally, some illustrative examples are included in the project github repository in the form of Jupyter Notebooks.

## 4 Conclusion

MEWpy offers a practical interface to several strain optimization heuristics, allowing to model and optimize microbial production on GSMMs defining gene–protein-reaction associations, but also on models enhanced with transcriptional and translational layers. Metaheuristics such as EAs and SA, including multi-objective methods, drive the optimization towards the best set of enzymes, genes or reactions, to under/over-express or delete to maximize the production of a target compound.

New methods are presently being added to enable CSO resorting to Metabolism Expression and Thermodynamics Flux (ETFL) models (Salvy and Hatzimanikatis, 2020) and Metabolism and Expression models (ME-models)(Lerman et al., 2012). By enabling the analysis and comparison of solutions obtained from distinct algorithms and modelling approaches, MEWpy will become an essential tool for the development of microbial cell factories towards the production of natural products.

## Funding

This project received funding from the European Union’s Horizon 2020 research and innovation programme [814408].

*Conflict of Interest*: none declared.
